# DAnIEL: A User-Friendly Web Server for Fungal ITS Amplicon Sequencing Data

**DOI:** 10.3389/fmicb.2021.720513

**Published:** 2021-08-17

**Authors:** Daniel Loos, Lu Zhang, Christine Beemelmanns, Oliver Kurzai, Gianni Panagiotou

**Affiliations:** ^1^Systems Biology and Bioinformatics Group, Leibniz Institute for Natural Product Research and Infection Biology, Jena, Germany; ^2^Chemical Biology of Microbe-Host Interactions Group, Leibniz Institute for Natural Product Research and Infection Biology, Jena, Germany; ^3^Institute for Hygiene and Microbiology, University of Würzburg, Würzburg, Germany; ^4^National Reference Center for Invasive Fungal Infections NRZMyk, Leibniz Institute for Natural Product Research and Infection Biology, Jena, Germany; ^5^Systems Biology and Bioinformatics Group, School of Biological Sciences, Faculty of Science, The University of Hong Kong, Pokfulam, China

**Keywords:** metagenomics, fungi, mycobiome, web server, ITS

## Abstract

Trillions of microbes representing all kingdoms of life are resident in, and on, humans holding essential roles for the host development and physiology. The last decade over a dozen online tools and servers, accessible *via* public domain, have been developed for the analysis of bacterial sequences; however, the analysis of fungi is still in its infancy. Here, we present a web server dedicated to the comprehensive analysis of the human mycobiome for (i) translating raw sequencing reads to data tables and high-standard figures, (ii) integrating statistical analysis and machine learning with a manually curated relational database and (iii) comparing the user’s uploaded datasets with publicly available from the Sequence Read Archive. Using 1,266 publicly available Internal transcribed spacers (ITS) samples, we demonstrated the utility of DAnIEL web server on large scale datasets and show the differences in fungal communities between human skin and soil sites.

## Introduction

Metagenomics provide a comprehensive view about microbial community structure. Previous studies have revealed many insights about the diversity, composition, and interaction patterns of bacterial communities. Fungi are a neglected but very important kingdom due to the important role they play in many human diseases (Mukherjee et al., 2014). The number of publications in PubMed related to the mycobiome is exponentially growing and increased more than 17-fold in the past 5years. Fungal metagenomics is becoming an essential part for comprehensive human host studies and should be accessible to the whole scientific community without the need of laborious and time-consuming efforts. We present DAnIEL (Describing, Analyzing and Integrating fungal Ecology to effectively study the systems of Life), the only web server that covers the whole workflow of ITS analysis beginning from raw reads to publication ready figures and tables, contains a relational database for the biological evaluation of statistical findings and allows comparative analysis with public available mycobiome datasets. For all steps, a summary of methods and results, including citations, is provided, and interactive plots can be created tailor-made. The web server is optimised to account for the properties of typical ITS datasets such as a high sparseness of the abundance profile and amplicon length variability. Whereas the web server can be used with ITS samples from all kinds of environments, we started to build the manual curated database with fungal species relevant for humans; however, many of the species are found in other environmental niches as well. DAnIEL is freely available at https://sbi.hki-jena.de/daniel.

## Design and Implementation

### Overview

**Figure 1:**
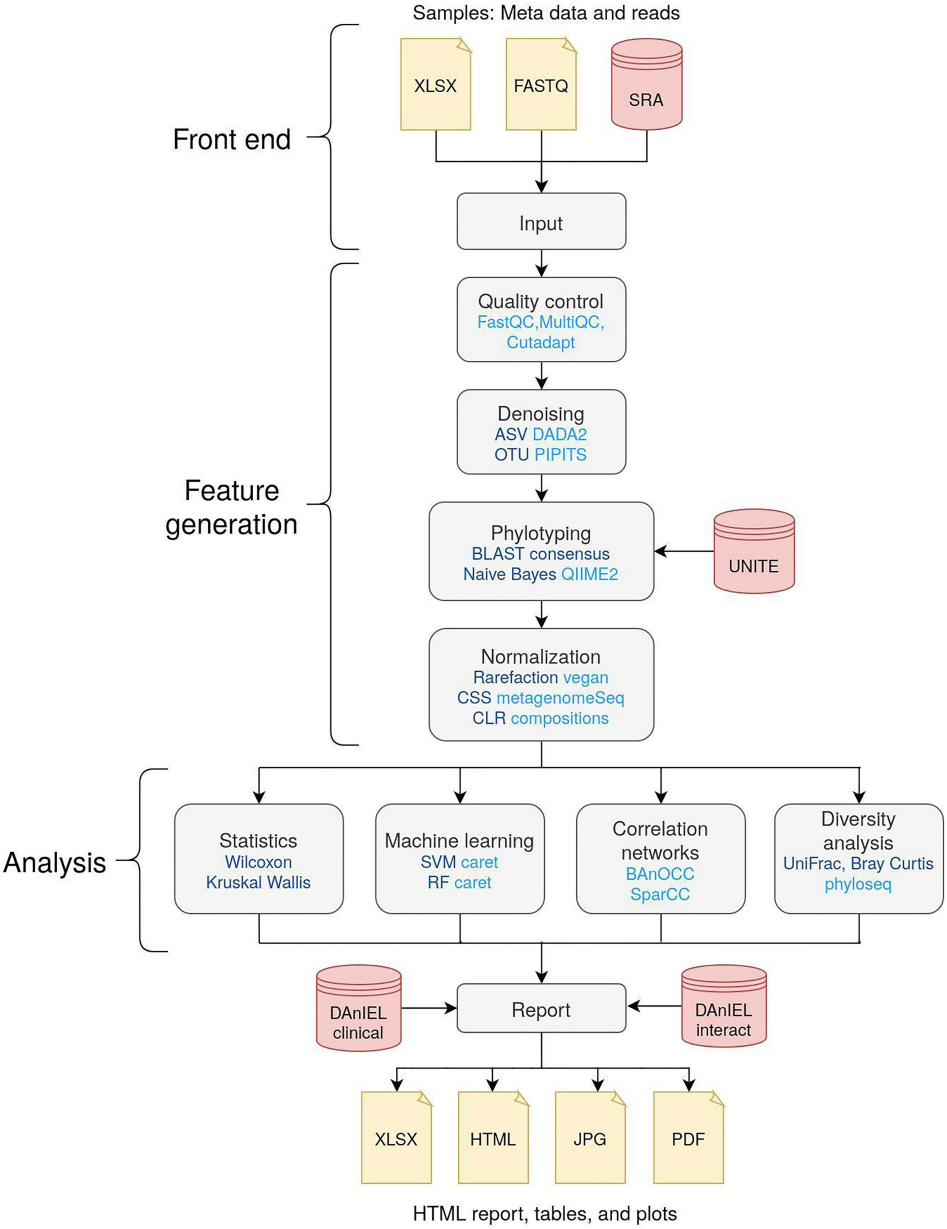
Overview of the workflow of the DAnIEL web server. Methods and tools are shown in dark and light blue, respectively. Databases are shown in red. Feature generation and analysis are part of the back end. Taxa are augmented using our relational database of clinical samples (DanIEL clinical) and interactions reported in the literature (DanIEL interact).

The workflow is illustrated in Figure 1. Raw reads can be uploaded in compressed FASTQ format. Optionally, read runs from the NCBI Sequence Read Archive (SRA) can be added by either selecting from the 700 existing cohorts of the DAnIEL database or by entering their accessions directly. Metadata about the samples can be uploaded in CSV or Excel file format if statistical analysis is needed. Parameter sets for tweaking the workflow can be created, e.g., to filter features by abundance or to trim custom primer sequences. A comprehensive documentation about parameters to tweak the workflow and a tutorial is available on the DAnIEL web server. To facilitate biological insights in significant features, we constructed a manually curated database containing 1,669 fungal interactions with diseases, bacteria species and immune components retrieved from 761 published papers. This database is used by the web server for biological interpretation of significantly different abundant or correlated taxa. Furthermore, we incorporated a list of clinical samples of species involved in fungal infections from the German National Reference Center for Invasive Fungal Infections (NRZMyk).

### Feature Generation

Features are generated from the raw reads provided. Samples are demultiplexed, if necessary, according to the barcode mapping provided in the metadata table. External samples are downloaded from the NCBI Sequence Read Archive using grabseqs (Taylor et al., 2020). Quality control (QC) is performed afterwards. FastQC and MultiQC are used to monitor sequencing errors (Ewels et al., 2016). Cutadapt is used to trim primer and adapter sequences (Martin, 2011). Samples can be excluded from downstream analysis using various criteria such as minimum number of quality-controlled reads or base quality tests specified by FastQC. Representative biological sequences are created from quality-controlled reads *via* denoising. Either OTUs or amplicon sequence variants (ASVs) can be called using PIPITS (Gweon et al., 2015) or DADA2 (Callahan et al., 2016), respectively. Taxonomy of denoised sequences is assigned using either Naive Bayes or BLAST consensus approach of QIIME2 (Bolyen et al., 2019). Abundance counts are pooled at any given taxonomic rank and filtered by abundance and prevalence. Lastly, pooled counts are normalised using the methods aware of different library sizes like rarefaction or cumulative sum scaling (CSS), as implemented in the R packages vegan and metagenomeSeq, respectively (Dixon, 2003; Paulson et al., 2013). Centered log-ratio (CLR) normalisation is used by default to account for the compositionality. The generated features are used in downstream analysis to infer biological insights.

### Relational Database Generation

DAnIEL was initially run on three cohorts to retrieve a list of fungal species relevant for analyses when studying human samples: Faecal samples from mycobiome datasets of cancer patients (*N*=71, ITS2, PRJEB33756; Mirhakkak et al., 2021), antibiotics intervention (*N*=59, ITS2, PRJNA579284; Seelbinder et al., 2020) and human skin swab samples (*N*=203, ITS1, PRJNA286273; Leung et al., 2016). For each species found, we constructed a NCBI Entrez query to search for PubMed abstracts. Terms “disease”, “cytokine”, “immune system” and “prokaryote” and a limit of 20 papers per species were used to narrow down the focus of our subsequent manual curation. In total 1,337 abstracts from these papers were reviewed to create a manually curated database of fungal interactions. Medical Subject Headings (MeSH) were used for annotations whenever applicable. In addition, FUNGuild was integrated to provide information about the trophic modes in an ecological context (Nguyen et al., 2016).

### Feature Analysis

Diversity is calculated using the R packages vegan (Dixon, 2003) and phyloseq (McMurdie and Holmes, 2013). Various methods, including principal coordinates analysis (PCoA) and non-metric multidimensional scaling (NMDS), can be used to generate ordination plots. FastSpar implementation of the SparCC algorithm can be used to create correlation networks of co-abundant taxa (Friedman and Alm, 2012; Watts et al., 2019). Alternatively, BAnOCC can be chosen to account for the compositionality of NGS abundance data (Schwager et al., 2017). The correlation analysis can be executed for each sample group individually, e.g., to compare networks of “case” and “control” samples. If a metadata table is provided, group-wise statistics are performed using Mann–Whitney U test for binary response variables and Kruskal–Wallis one-way analysis of variance in combination with Dunn’s *post hoc* test (Dunn, 1964) for other nominal responses. Spearman’s rank correlation is used for continuous responses instead. Features significant in any of these tests are annotated with our manually curated database of fungal interactions and clinical samples. Machine learning is applied to categorical response variables using the R package caret (Kuhn, 2008). Both random forest (RF) and support vector machines (SVMs) are used in combination with ANOVA filter and recursive feature selection. Best performing models according to the area under the receiver operating curve (AUC) in 5-fold cross-validation and feature importance scores are reported.

### Technical Design

The overall pipeline of the DAnIEL web server consists of two parts: A front-end the user is interacting with to upload and visualise the data and a back-end workflow responsible for processing the uploaded data. The front-end of DAnIEL web server is implemented as an R shiny app. For visualisation ggplot2 is used (Wickham et al., 2019) and Rmarkdown to create summary reports. The back-end is built as a Snakemake workflow (Koster and Rahmann, 2012). This allows running the workflow separately on any Linux system including computing clusters. Conda is used to create reproducible environments for installing and running scripts and individual tools. A unique identifier will be assigned to each project to access the results later on. This also acts as a token for authentication. The tutorial consisting of 38 samples usually takes approximately half an hour wall time using 10 threads to be fully processed. Reports and visualisations can be accessed at the front-end once the corresponding step in the workflow has finished. This includes interactive plots and a summary consisting of findings, annotations, methods and references in a single HTML file.

## Results

### Comparison to Relevant Softwares

An overview on related software packages for analysing fungal amplicon sequencing data is given in Table 1. QIIME2 is a command-line focused tool; therefore, it is not ideal for researchers without programming skills (Bolyen et al., 2019). ITScan covers profiling of operational taxonomic units (OTUs), however it does not cover quality control of raw reads (Ferro et al., 2014). CloVR-ITS was designed for pyrosequencing data; whereas DAnIEL is built for illumina paired-end data (White et al., 2013). Most tools are lacking the ability to calculate correlation networks especially those aware of the compositional nature of taxon counts, which is crucial in most analyses (Gloor et al., 2017). Tools like PipeCraft and LotuS focus on calculating the OTU table (Hildebrand et al., 2014; Anslan et al., 2017). Many existing tools are general and do not account for properties of a typical ITS dataset by default. For example, the length of ITS1 can range from 9 to 1,181bp (Yang et al., 2018). We chose 50bp as the default minimal QC read length as a trade-off to be able to detect fungi with a short ITS region while still have enough bases left for an accurate taxonomic classification. To the best of our knowledge, DAnIEL is the only web server available covering the whole workflow of ITS analysis beginning from raw reads to publication ready figures and tables, as well as, integration with a relational database for biological evaluation of statistical findings and comparative analysis with public available mycobiome data sets.

**Table 1:** Functionality of software for ITS analysis.

		DAnIEL	QIIME2	mothur	CloVR-ITS	ITScan	SEED2	PipeCraft	LotuS
Usabilty	Web server	+	−	−	−	+	−	−	−
	GUI	+	−	+	+	+	+	+	−
	HTML report	+	+	−	−	+	−	−	−
Data	Additional cohorts	+	−	−	−	−	−	−	−
	ITS tailored	+	−	−	+	+	−	−	−
Profiling	Quality control	+	+	+	+	−	+	+	+
	OTU profiling	+	+	+	+	+	+	+	+
	ASV profiling	+	+	−	−	−	−	−	+
Analysis	Diversity	+	+	+	+	+	+	−	−
	SparCC correlation	+	+	+	−	−	−	−	−
	BAnOCC correlation	+	−	−	−	−	−	−	−
	Machine Learning	+	+	+	−	−	−	−	−
	Knowledge base	+	−	−	−	−	−	−	−

### Case Studies

**Figure 2:**
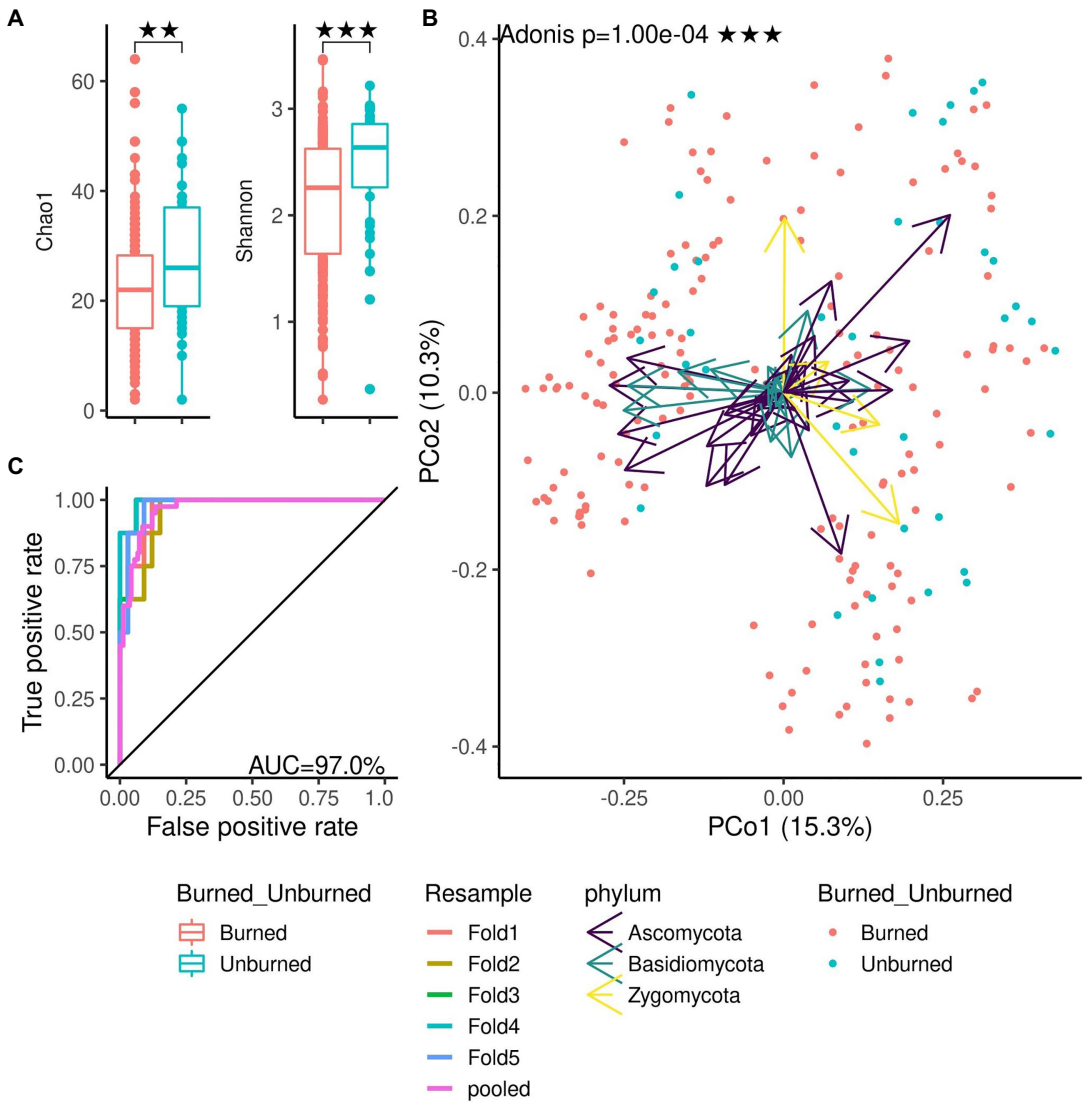
Mycobiome comparison of burned and unburned soil samples. All figures were directly generated by the web server. **(A)** Alpha diversity. **(B)** Beta diversity: Ordination of Bray-Curtis dissimilarities. **(C)** Area under ROC in predicting the burning site from the abundance profile (best model, random forest). ^**^p < 0.01, ^***^p < 0.001.

**Figure 3:**
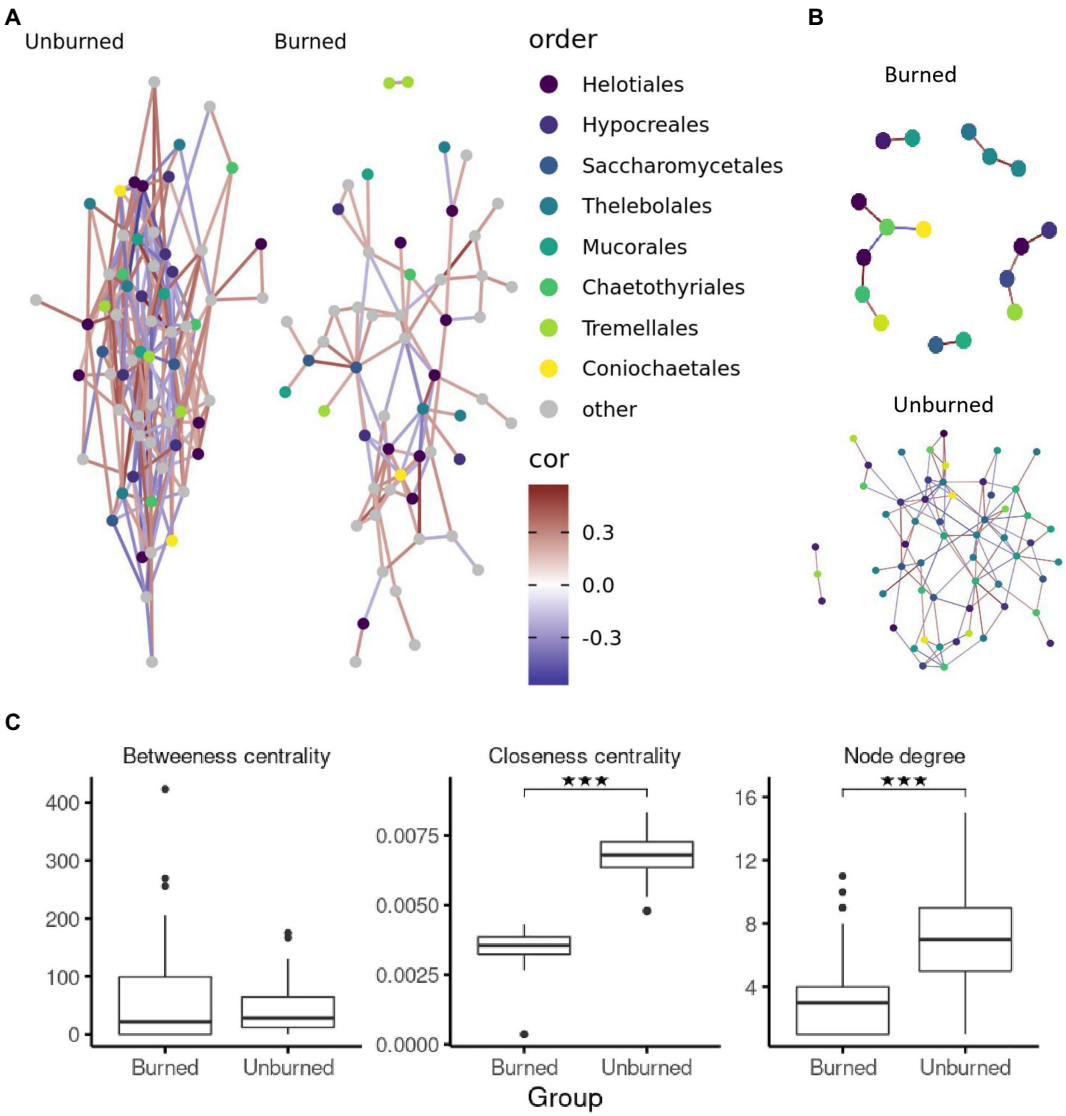
Correlation networks of burned and unburned soil samples. SparCC correlation networks per sample group using **(A)** default threshold $\left(\left|r\right|>0.2\right)$ and (**B**; $\left|r\right|>0.3$) using the interactive GUI. **(C)** Distribution of network topology metrics over genera in the correlation network. ^***^p < 0.001.

We demonstrated the functionality of the DAnIEL web server by running it on public available cohorts of soil and human mycobiomes. The first cohort investigated the effects of wildfire on the soil fungi in northwestern Canadian boreal forest (*N*=300, NCBI Accession PRJNA564811; Whitman et al., 2019). The cohort was selected from the integrated database of fungal projects. DAnIEL was run with default parameters. The results are shown in Figures 2, 3. All sub figures were directly generated by the web server. We confirmed that fungal communities were strongly dissimilar between burned and unburned sites. Burned sites showed significantly decreased Shannon and Chao1 alpha diversity metrics (Wilcoxon rank sum test, *p*<0.01). Fungal Bray-Curtis dissimilarities differ also significantly (Adonis PERMANOVA, *p*<10^−4^). Furthermore, a disrupted co-abundance pattern was observed in burned sites using SparCC correlation networks. Genus node degree and betweenness centrality are significantly decreased (Wilcoxon rank sum test, *p*<0.01). We increased the minimal absolute correlation coefficient to ${\left|r\right|}_{\min,SparCC}=0.3$, in the interactive network exploration of DAnIEL to emphasise this coabundance fragmentation. A Random Forest was picked to be the best model in predicting the state (burned vs. unburned) based on the fungal abundance profile (AUC=98% in 5-fold cross validation).

Secondly, we performed a meta-analysis of two publicly available human skin mycobiomes about dandruff (*N*=966, ITS1, PRJNA415710; Saxena et al., 2021) and chronic wounds (*N*=384, ITS1, PRJNA324668; Kalan et al., 2018). Results of the human case study are shown in Supplementary Figures 1, 2. Wound sites showed significantly decreased Shannon and Chao1 alpha diversity metrics (Wilcoxon, *p*<0.001). Fungal Bray-Curtis dissimilarities differ also significantly (Adonis PERMANOVA, p<10^−4^). Wound samples showed increased abundances in *Malassezia* and *Saccharomyces*. A fungal co-abundance network was only possible to be constructed for dandruff samples after filtering with default parameters. The minimum absolute SparCC correlation coefficient was lowered to 0.1 to check the robustness of the networks. This revealed three co-abundant genera pairs in the wound samples, but this network was still much sparser than the network obtained from dandruff samples. A random forest model was picked to be the best one in predicting the skin type based on the fungal abundance profile (AUC=99% in 5-fold cross validation) with *Saccharomyces* and *Ascomycota* spp. Showing high Gini feature importance. The higher AUC value compared to the soil example indicated a stronger non-linear biological signal discriminating the sample groups using the fungal abundance profile.

Taxa found significant in either of differential abundance or co-abundance analysis were annotated with FUNGuild (Nguyen et al., 2016) and our integrated relational databases. The genera found significantly different in the human cohort were assigned with 867 interactions to other bacteria or cytokines and 141 infection related samples from our manually curated database (Supplementary Figure 3). The number of annotations varied across the clades according to their coverage of published literature. For the soil cohort, however, the genera found significant showed only 214 interactions reported in the literature and 25 infection related samples being reported confirming the human focus of our database.

### Benchmarking

Processing durations were benchmarked on a Docker container provided with 10 cores and 100GB of RAM. It took 2.9h to process the soil samples and 62.9h to process the human samples (see Supplementary Figure 4). The time limiting step in big cohorts of the pipeline is the denoising process, which can be parallelised much better for ASV profiling compared to OTU profiling. Downloading and quality control takes usually less than a minute per sample.

The performance of taxonomic profiling was evaluated using simulated reads. Grinder was used to simulate 10 samples in which each of them consists of $500\times 10^{3}$ 150bp paired-end (PE) reads (Angly et al., 2012). Primers ITS1 and ITS2 targeting the ITS1 sub region were utilised to simulate abundances from 100 different reference sequences following an exponential distribution. The same database UNITE 8.2 dynamic was used for simulation and training the classifiers to enable a fair comparison (Nilsson et al., 2019). To simulate biological variability, a uniform mutation rate of 1% was incorporated, in which substitutions were four times more likely than insertions or deletions. DAnIEL was run on the data with different methods for denoising (DADA2 and PIPITS) and taxonomic classification (BLAST consensus and Naive Bayes). Benchmarking performance of profiling abundance was based on (Ye et al., 2019) using counts pooled at genus rank. Briefly, the Euclidean distance (L2 norm) between the measured and the true abundance profile was calculated for each sample. Furthermore, differences of abundances were calculated for each sample and taxon separately. Pure taxon occurrence was evaluated by counting samples, in which a taxon was both measured and simulated. Precision, Sensitivity, Specificity and F1 score were calculated based on this contingency table. Taxon occurrences were very specifically but less sensitively profiled (98 and 33% on average, respectively, see Supplementary Figure 5). DADA2 outperformed PIPITS in all metrics. The outperformance of using ASV compared to OTU is consistent with the literature (Callahan et al., 2017; Caruso et al., 2019). ASVs are more accurate and allow a more detailed analysis of the mycobiome studied. However, this can be also disadvantageous. The detailed taxonomic profile of ASVs in taxonomically diverse studies like environmental samples can make a manual curation of sequence alignments and statistical downstream analyses more difficult. Furthermore, intragenomic variation can result in multiple ASVs originating from the same fungal cell overestimating the true diversity (Schoch et al., 2012). On the other hand, OTU profiling is less sensitive to potentially unwanted details, and we still considered it as a necessary function in DAnIEL to make the results comparable with older studies.

Naive Bayes classification outperformed the BLAST consensus approach in terms of specificity and precision but not in sensitivity. Most accurate abundance profiles were generated using DADA2 (see Supplementary Figure 6). PIPITS underestimated many abundances, which lead to increased distances in some samples especially in combination with the Naive Bayes classifier. The values were very similar for different phyla. Therefore, taxonomy seemed to have only little influence on the classification performance.

Furthermore, we compared the results in the case studies using both ASV and OTU profiling. The results are shown in Supplementary Figures 7, 8. Most of the high abundant taxa were found using any denoising method with similar abundance values and correlation networks. The alpha diversity was significantly higher using OTU profiling (Wilcoxon rank sum test, *p*<0.001).

### Relational Database Generalisability

We evaluated the generalisability of the DAnIEL interactions database on 30 other ITS studies from various habitats. The results are shown in Supplementary Figure 9. Only genera prevalent in at least 10% of the samples in any habitat were considered for this analysis. On average, 29% of the prevalent genera in host habitats, 28% in aquatic and 15% in soil samples were already found in our manually curated database.

## Availability and Future Directions

DAnIEL is freely available as a web service at https://sbi.hki-jena.de/daniel. There is no registration required. Instead, an ID token will be assigned to each project. Results will be available for 30days. The source code is hosted at https://github.com/bioinformatics-leibniz-hki/DAnIEL, together with several tests and an example on how to use it, and is distributed under the BSD-2-Clause license. All databases including reference sequences, existing cohorts and fungal interactions can be downloaded at https://doi.org/10.5281/zenodo.4073125.

Since sequencing costs dropped drastically in the past decade, whole metagenome sequencing (WMS) becomes more and more popular. This web server for amplicon sequencing, however, will still be relevant in the future, because many large reference cohorts including the American Gut Project and the Earth Microbiome Project are based on ITS sequencing and one needs a very high sample size to conduct machine learning and correlation network analyses (Thompson et al., 2017; McDonald et al., 2018). Furthermore, amplicon sequencing can be helpful in identifying low abundant fungi.

Using the Snakemake workflow engine, DAnIEL can be easily extended by other steps (Koster and Rahmann, 2012). For instance, Picrust2 can be integrated to predict fungal function profiles (Douglas et al., 2020). Fungal taxonomic profiling profits from sequencing larger amplicons. Therefore, tools specifically designed for long read sequencing data of the third generation can be used for quality control to improve classification performance. Furthermore, updating the manually curated database and augmenting it with text mining approaches will improve the biological interpretation of significant taxa.

## Data Availability Statement

The datasets presented in this study can be found in online repositories. The names of the repository/repositories and accession number(s) can be found in the article/Supplementary Material.

## Author Contributions

DL and GP conceived the study, designed the web server, and wrote the manuscript. DL implemented the web server. LZ and CB curated the relational database of fungal interactions. OK developed the NRZMyk database. DL processed the existing projects. All authors contributed to the article and approved the submitted version.

## Conflict of Interest

The authors declare that the research was conducted in the absence of any commercial or financial relationships that could be construed as a potential conflict of interest.

## Publisher’s Note

All claims expressed in this article are solely those of the authors and do not necessarily represent those of their affiliated organizations, or those of the publisher, the editors and the reviewers. Any product that may be evaluated in this article, or claim that may be made by its manufacturer, is not guaranteed or endorsed by the publisher.

## References

[ref1] Angly F. E. Willner D. Rohwer F. Hugenholtz P. Tyson G. W. (2012). Grinder: a versatile amplicon and shotgun sequence simulator. Nucleic Acids Res. 40:e94. 10.1093/nar/gks251, PMID: 22434876 PMC3384353

[ref2] Anslan S. Bahram M. Hiiesalu I. Tedersoo L. (2017). PipeCraft: flexible open-source toolkit for bioinformatics analysis of custom high-throughput amplicon sequencing data. Mol. Ecol. Resour. 17, e234–e240. 10.1111/1755-0998.12692, PMID: 28544559

[ref3] Bolyen E. Rideout J. R. Dillon M. R. Bokulich N. A. Abnet C. C. Al-Ghalith G. A.. (2019). Reproducible, interactive, scalable and extensible microbiome data science using QIIME 2. Nat. Biotechnol. 37, 852–857. 10.1038/s41587-019-0209-9, PMID: 31341288 PMC7015180

[ref4] Callahan B. J. McMurdie P. J. Holmes S. P. (2017). Exact sequence variants should replace operational taxonomic units in marker-gene data analysis. ISME J. 11, 2639–2643. 10.1038/ismej.2017.119, PMID: 28731476 PMC5702726

[ref5] Callahan B. J. McMurdie P. J. Rosen M. J. Han A. W. Johnson A. J. A. Holmes S. P. (2016). DADA2: high-resolution sample inference from illumina amplicon data. Nat. Methods 13, 581–583. 10.1038/nmeth.3869, PMID: 27214047 PMC4927377

[ref6] Caruso V. Song X. Asquith M. Karstens L. (2019). Performance of microbiome sequence inference methods in environments with varying biomass. mSystems 4:e00163–18. 10.1128/mSystems.00163-18, PMID: 30801029 PMC6381225

[ref7] Dixon P. (2003). VEGAN, a package of r functions for community ecology. J. Veg. Sci. 14, 927–930. 10.1111/j.1654-1103.2003.tb02228.x

[ref8] Douglas G. M. Maffei V. J. Zaneveld J. R. Yurgel S. N. Brown J. R. Taylor C. M.. (2020). PICRUSt2 for prediction of metagenome functions. Nat. Biotechnol. 38, 685–688. 10.1038/s41587-020-0548-6, PMID: 32483366 PMC7365738

[ref9] Dunn O. J. (1964). Multiple comparisons using rank sums. Technometrics 6, 241–252. 10.1080/00401706.1964.10490181

[ref10] Ewels P. Magnusson M. Lundin S. Käller M. (2016). MultiQC: summarize analysis results for multiple tools and samples in a single report. Bioinformatics 32, 3047–3048. 10.1093/bioinformatics/btw354, PMID: 27312411 PMC5039924

[ref11] Ferro M. Antonio E. A. Souza W. Bacci M. (2014). ITScan: a web-based analysis tool for Internal Transcribed Spacer (ITS) sequences. BMC Res. Notes 7:857. 10.1186/1756-0500-7-857, PMID: 25430816 PMC4258023

[ref12] Friedman J. Alm E. J. (2012). Inferring correlation networks from genomic survey data. PLoS Comput. Biol. 8:e1002687. 10.1371/journal.pcbi.1002687, PMID: 23028285 PMC3447976

[ref13] Gloor G. B. Macklaim J. M. Pawlowsky-Glahn V. Egozcue J. J. (2017). Microbiome datasets are compositional: And This is not optional. Front. Microbiol. 8:2224. 10.3389/fmicb.2017.02224, PMID: 29187837 PMC5695134

[ref14] Gweon H. S. Oliver A. Taylor J. Booth T. Gibbs M. Read D. S.. (2015). PIPITS: an automated pipeline for analyses of fungal internal transcribed spacer sequences from the illumina sequencing platform. Methods Ecol. Evol. 6, 973–980. 10.1111/2041-210X.12399, PMID: 27570615 PMC4981123

[ref15] Hildebrand F. Tadeo R. Voigt A. Y. Bork P. Raes J. (2014). LotuS: an efficient and user-friendly OTU processing pipeline. Microbiome 2:30. 10.1186/2049-2618-2-30, PMID: 27367037 PMC4179863

[ref16] Kalan L. Meisel J. S. Loesche M. A. Horwinski J. Soaita I. Chen X.. (2018). The microbial basis of impaired wound healing: differential roles for pathogens, “bystanders”, and strain-level diversification in clinical outcomes. bioRxiv [Preprint]. 10.1101/427567.X

[ref17] Koster J. Rahmann S. (2012). Snakemake—a scalable bioinformatics workflow engine. Bioinformatics 28, 2520–2522. 10.1093/bioinformatics/bts480, PMID: 22908215

[ref18] Kuhn M. (2008). Building predictive models in r using the caret package. J. Stat. Softw. 28, 1–26. 10.18637/jss.v028.i0527774042

[ref19] Leung M. H. Y. Chan K. C. K. Lee P. K. H. (2016). Skin fungal community and its correlation with bacterial community of urban chinese individuals. Microbiome 4:46. 10.1186/s40168-016-0192-z, PMID: 27558504 PMC4997687

[ref20] Martin M. (2011). Cutadapt removes adapter sequences from high-throughput sequencing reads. EMBnet. J. 17, 10–12. 10.14806/ej.17.1.200

[ref21] McDonald D. Hyde E. Debelius J. W. Morton J. T. Gonzalez A. Ackermann G.. (2018). American gut: an open platform for citizen science microbiome research. mSystems 3:e00031–18. 10.1128/mSystems.00031-18, PMID: 29795809 PMC5954204

[ref22] McMurdie P. J. Holmes S. (2013). Phyloseq: an R package for reproducible interactive analysis and graphics of microbiome census data. PLoS One 8:e61217. 10.1371/journal.pone.0061217, PMID: 23630581 PMC3632530

[ref23] Mirhakkak M. H. Schäuble S. Klassert T. E. Brunke S. Brandt P. Loos D.. (2021). Metabolic modeling predicts specific gut bacteria as key determinants for Candida albicans colonization levels. ISME J. 15, 1257–1270. 10.1038/s41396-020-00848-z, PMID: 33323978 PMC8115155

[ref24] Mukherjee J. A. R. Pranab K. Chandra (2014). Oral mycobiome analysis of HIV-infected patients: identification of pichia as an antagonist of opportunistic fungi. PLoS Pathog. 10:e1003996. 10.1371/journal.ppat.1003996, PMID: 24626467 PMC3953492

[ref25] Nguyen N. H. Song Z. Bates S. T. Branco S. Tedersoo L. Menke J.. (2016). FUNGuild: an open annotation tool for parsing fungal community datasets by ecological guild. Fungal Ecol. 20, 241–248. 10.1016/j.funeco.2015.06.006

[ref26] Nilsson R. H. Larsson K.-H. Taylor A. F. S. Bengtsson-Palme J. Jeppesen T. S. Schigel D.. (2019). The UNITE database for molecular identification of fungi: handling dark taxa and parallel taxonomic classifications. Nucleic Acids Res. 47, D259–D264. 10.1093/nar/gky1022, PMID: 30371820 PMC6324048

[ref27] Paulson J. N. Stine O. C. Bravo H. C. Pop M. (2013). Differential abundance analysis for microbial marker-gene surveys. Nat. Methods 10, 1200–1202. 10.1038/nmeth.2658, PMID: 24076764 PMC4010126

[ref28] Saxena R. Mittal P. Clavaud C. Dhakan D. B. Roy N. Breton L.. (2021). Longitudinal study of the scalp microbiome suggests coconut oil to enrich healthy scalp commensals. Sci. Rep. 11:7220. 10.1038/s41598-021-86454-1, PMID: 33790324 PMC8012655

[ref29] Schoch C. L. Seifert K. A. Huhndorf S. Robert V. Spouge J. L. Levesque C. A.. (2012). Nuclear ribosomal internal transcribed spacer (ITS) region as a universal DNA barcode marker for fungi. Proc. Natl. Acad. Sci. 109, 6241–6246. 10.1073/pnas.1117018109, PMID: 22454494 PMC3341068

[ref30] Schwager E. Mallick H. Ventz S. Huttenhower C. (2017). A bayesian method for detecting pairwise associations in compositional data. PLoS Comput. Biol. 13:e1005852. 10.1371/journal.pcbi.1005852, PMID: 29140991 PMC5706738

[ref31] Seelbinder B. Chen J. Brunke S. Vazquez-Uribe R. Santhaman R. Meyer A. C.. (2020). Antibiotics create a shift from mutualism to competition in human gut communities with a longer-lasting impact on fungi than bacteria. Microbiome 8:133. 10.1186/s40168-020-00899-6, PMID: 32919472 PMC7488854

[ref32] Taylor L. J. Abbas A. Bushman F. D. (2020). Grabseqs: simple downloading of reads and metadata from multiple next-generation sequencing data repositories. Bioinformatics 36, 3607–3609. 10.1093/bioinformatics/btaa167, PMID: 32154830 PMC7267817

[ref33] Thompson L. R. Sanders J. G. McDonald D. Amir A. Ladau J. Locey K. J.. (2017). A communal catalogue reveals Earth’s multiscale microbial diversity. Nature 551, 457–463. 10.1038/nature24621, PMID: 29088705 PMC6192678

[ref34] Watts S. C. Ritchie S. C. Inouye M. Holt K. E. (2019). FastSpar: rapid and scalable correlation estimation for compositional data. Bioinformatics 35, 1064–1066. 10.1093/bioinformatics/bty734, PMID: 30169561 PMC6419895

[ref35] White J. R. Maddox C. White O. Angiuoli S. V. Fricke W. F. (2013). CloVR-ITS: automated internal transcribed spacer amplicon sequence analysis pipeline for the characterization of fungal microbiota. Microbiome 1:6. 10.1186/2049-2618-1-6, PMID: 24451270 PMC3869194

[ref36] Whitman T. Whitman E. Woolet J. Flannigan M. D. Thompson D. K. Parisien M.-A. (2019). Soil bacterial and fungal response to wildfires in the Canadian boreal forest across a burn severity gradient. Soil Biol. Biochem. 138:107571. 10.1016/j.soilbio.2019.107571

[ref37] Wickham H. Averick M. Bryan J. Chang W. McGowan L. François R.. (2019). Welcome to the tidyverse. J. Open Source Softw. 4:1686. 10.21105/joss.01686

[ref38] Yang R. H. Su J. H. Shang J. J. Wu Y. Y. Li Y. Bao D. P.. (2018). Evaluation of the ribosomal DNA internal transcribed spacer (ITS), specifically ITS1 and ITS2, for the analysis of fungal diversity by deep sequencing. PLoS One 13:e0206428. 10.1371/journal.pone.0209775, PMID: 30359454 PMC6201957

[ref39] Ye S. H. Siddle K. J. Park D. J. Sabeti P. C. (2019). Benchmarking metagenomics tools for taxonomic classification. Cell 178, 779–794. 10.1016/j.cell.2019.07.010, PMID: 31398336 PMC6716367

