# Supplementary material for: DAnIEL: A User-Friendly Web Server for Fungal ITS Amplicon Sequencing Data
Source: Front Microbiol. 2021 Aug 17;12:720513. doi: 10.3389/fmicb.2021.720513 (PMC8416086; doi:10.3389/fmicb.2021.720513)
Supplement: Supplementary file 1 [file Presentation_1.pdf]

Supplementary Information

DAnIEL: a user-friendly web server for fungal ITS amplicon sequencing data

Daniel Loos[1], Lu Zhang[1], Christine Beemelmanns[2], Oliver Kurzai[3], Gianni Panagiotou[1,4,*]

[1]Systems Biology and Bioinformatics, Leibniz Institute for Natural Product Research and Infection Biology, Germany, [2]Chemical Biology of Microbe-Host Interactions, Leibniz Institute for Natural Product Research and Infection Biology, Germany, [3]Institute for Hygiene and Microbiology, University of Würzburg, Germany, [4]Systems Biology and Bioinformatics, School of Biological Sciences, Faculty of Science, The University of Hong Kong, China

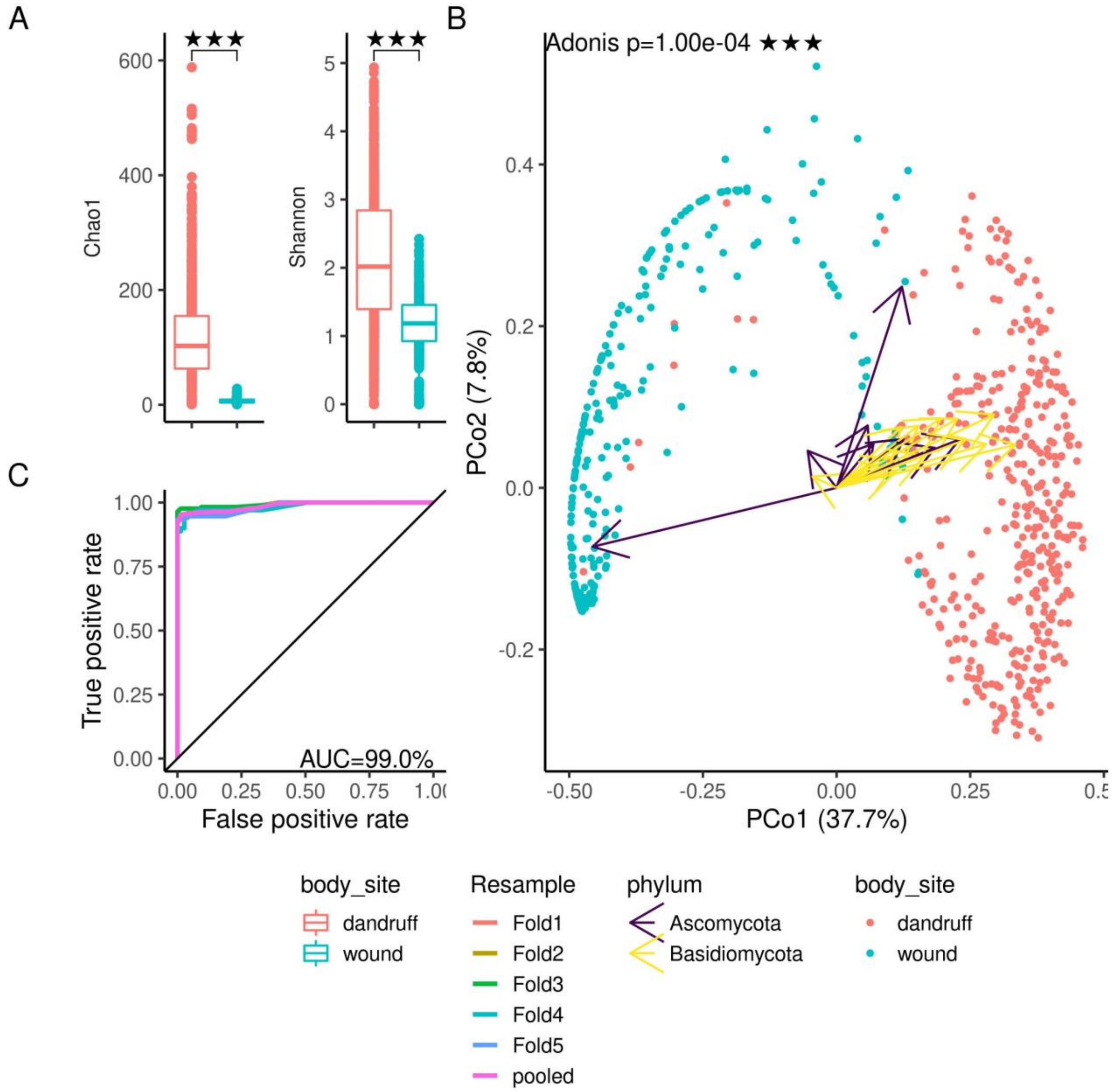

Fig. S1: Mycobiome comparison of the human skin (dandruff vs wound) using DAnIEL. All figures were directly generated by the web server. **A**: Alpha diversity. **B**: Beta diversity: Ordination of Bray-Curtis dissimilarities. **C**: Area under ROC in predicting the burning site from the abundance profile (best model, random forest)

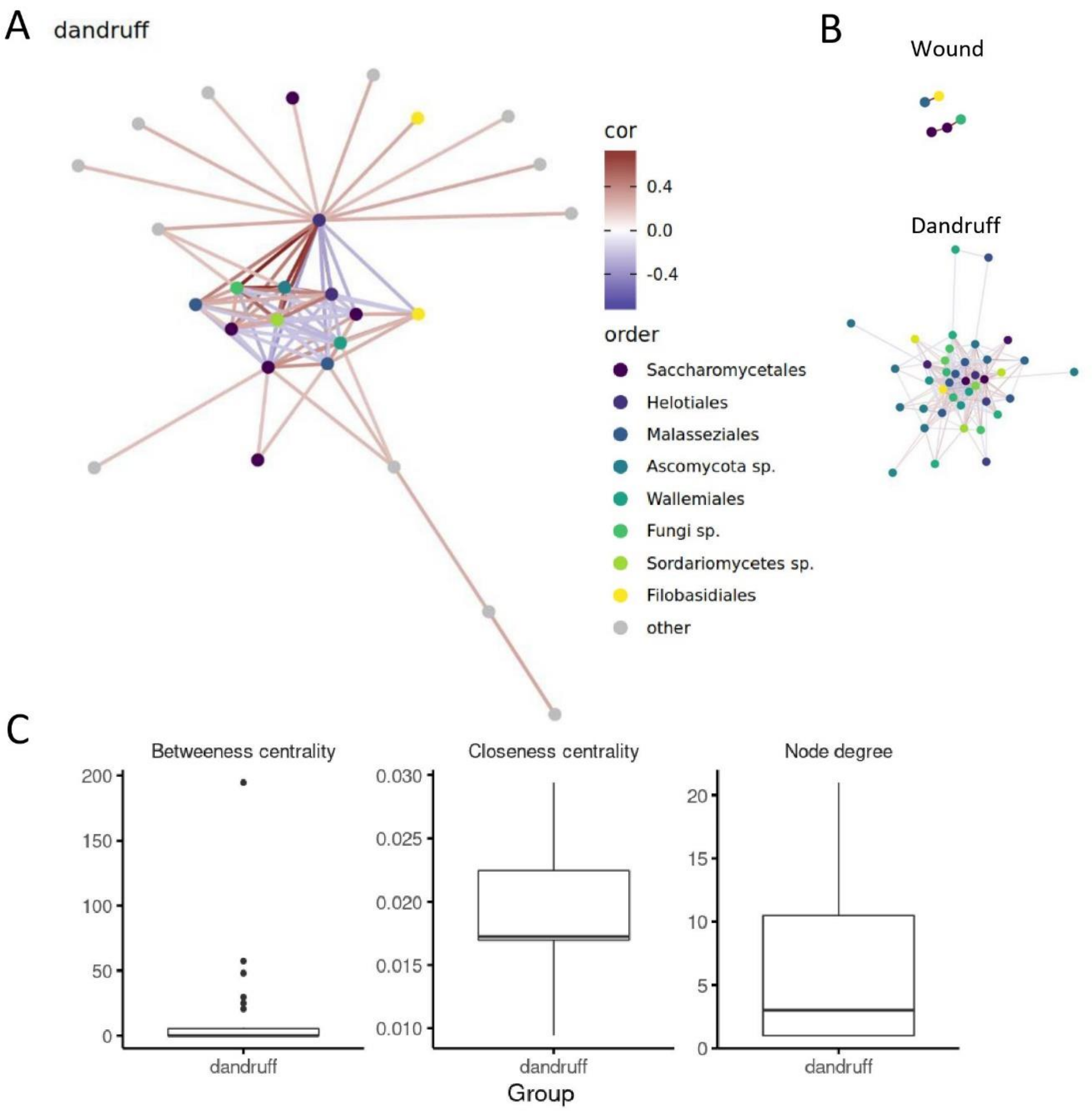

Fig S2: . Correlation networks of wound and dandruff humansamples. SparCC correlation networks per sample group using **A:** default threshold ($|r| > 0.2$) and **B:** ($|r| > 0.1$) using the interactive GUI. **C**: Distribution of network topology metrics over genera in the correlation network.

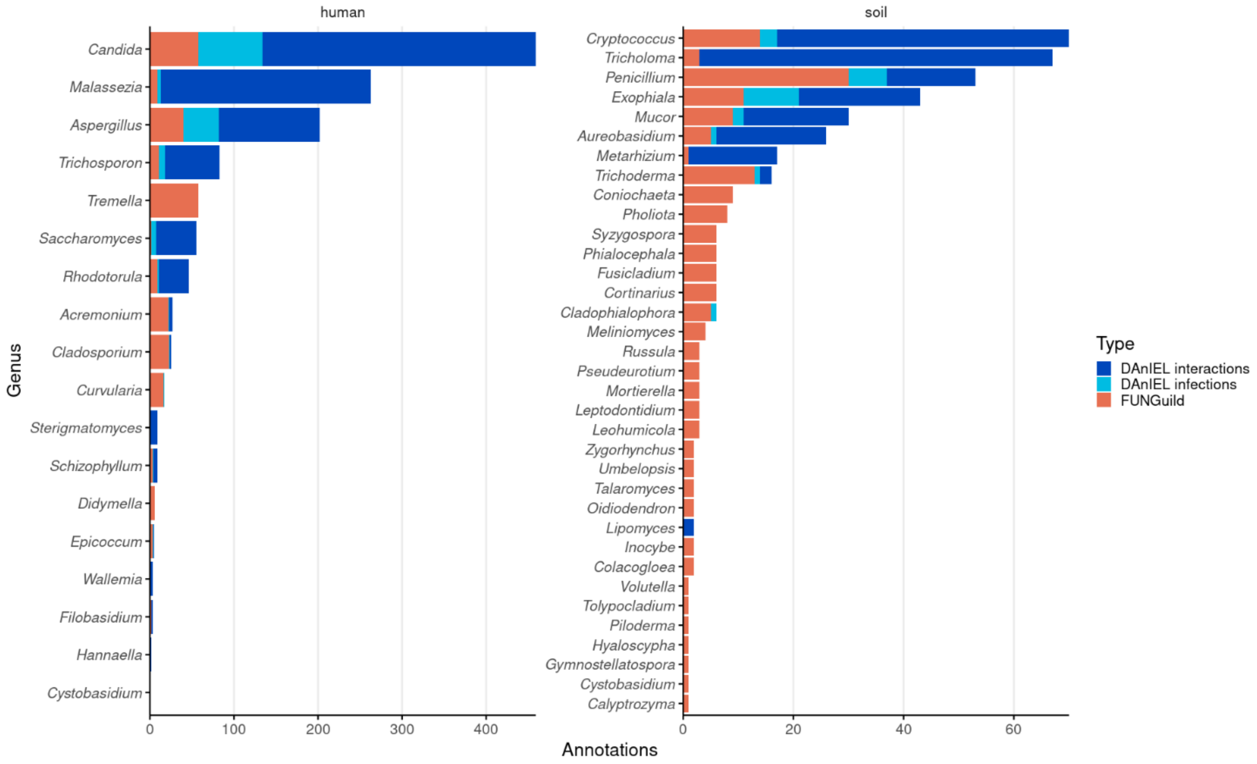

Fig. S3: Annotations of the integrated databases of manually curated fungal interactions, infected clinical samples and FUNGuild by cohort of the case study.

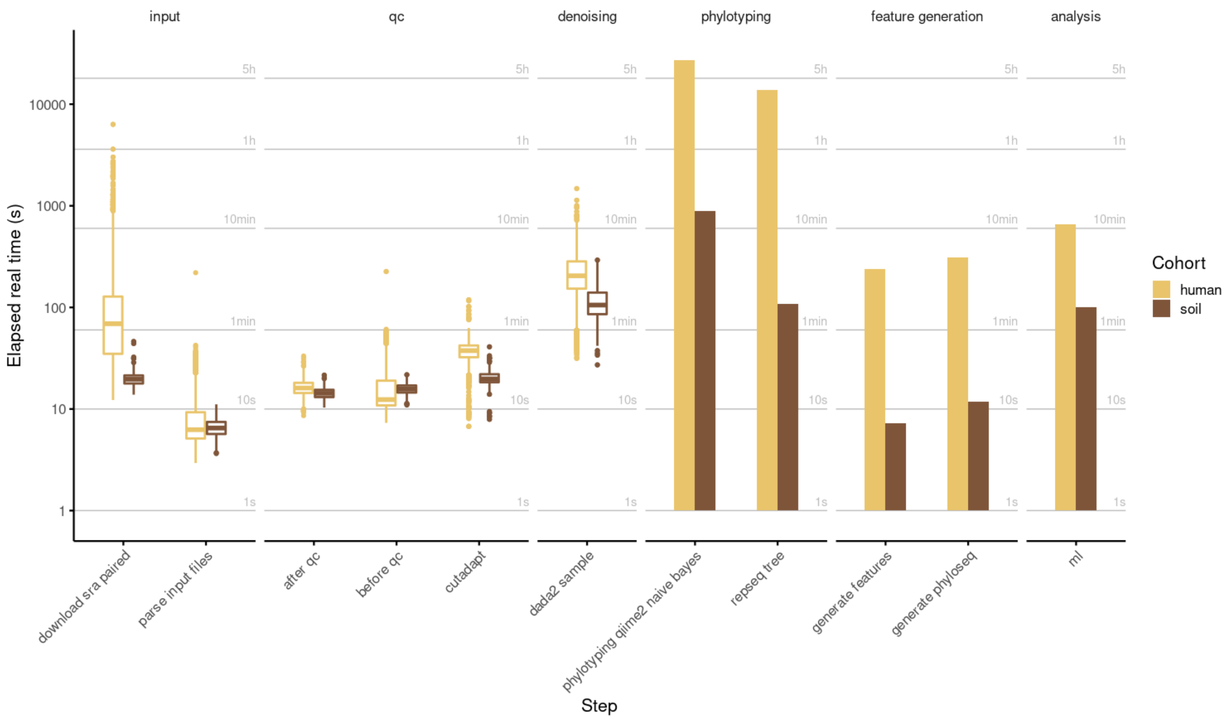

Fig. S4: Benchmarking processing durations. Elapsed real time was measured for each step of the workflow. Boxplots are shown if the steps were run in parallel for each sample. It took 2.9 h to process the soil samples (N=300) and 62.9 h to process the human samples (N=1350).

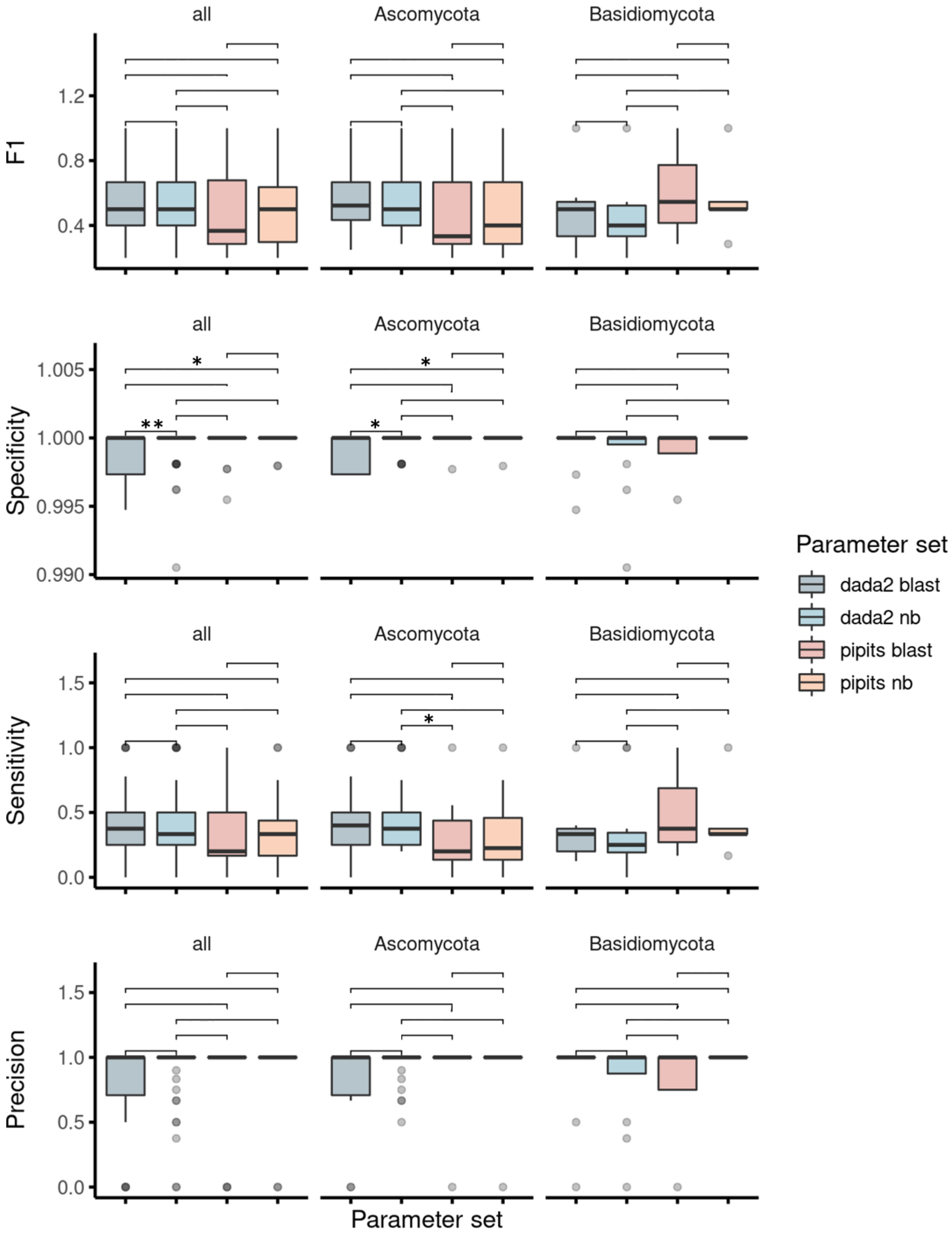

Fig. S5: Benchmarking taxon existence. DAnIEL was run on 10 samples consisting simulated reads using denoising methods DADA2 and PIPITS and classification methods BLAST consensus (blast) and Naïve Bayes (nb). Contingency tables were calculated by counting samples in which a taxon was both measured and simulated. DADA2 outperformed PIPITS in all metrics. NB classification outperformed BLAST in terms of specificity and precision but not in sensitivity.

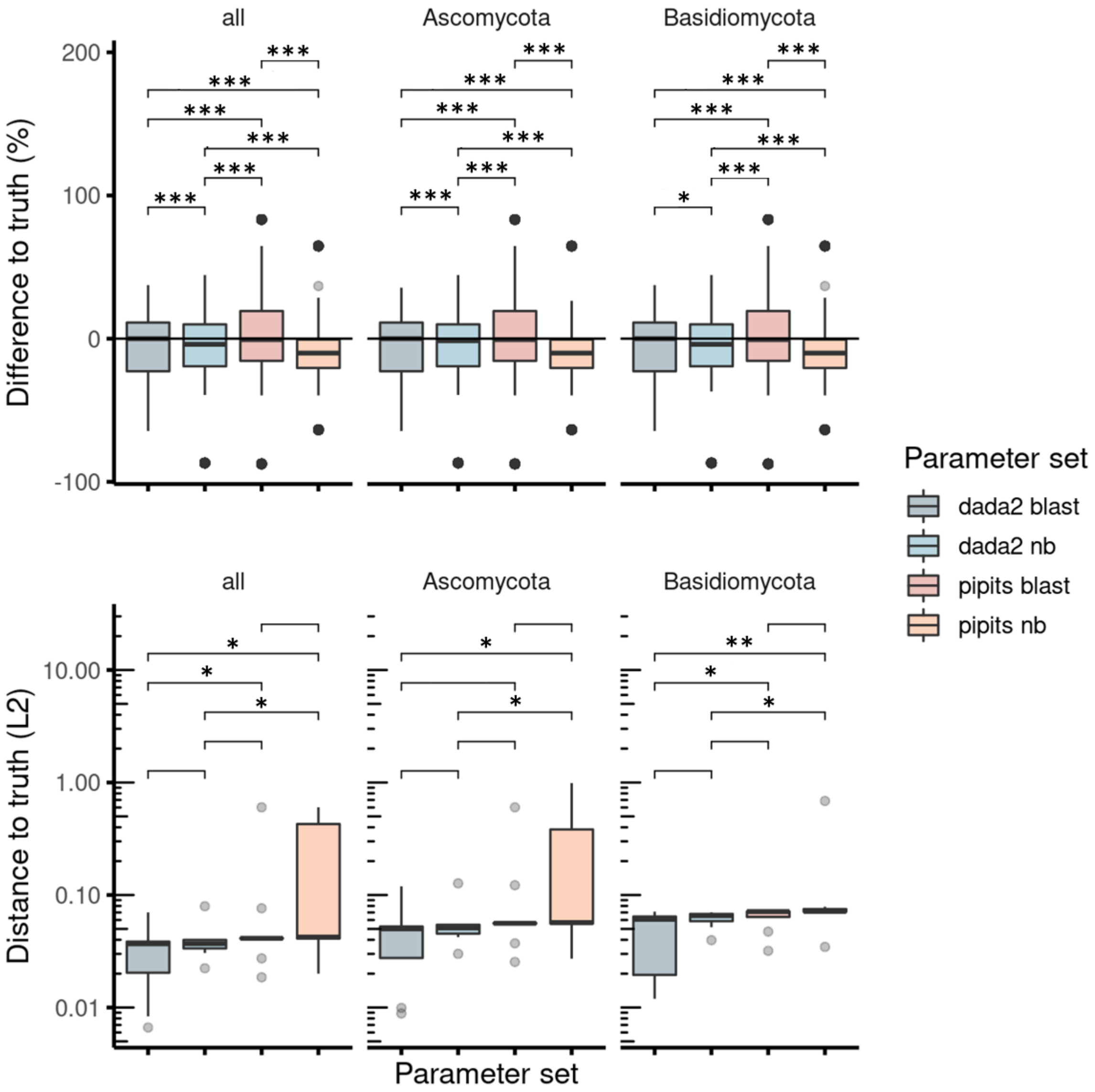

Fig. S6: Benchmarking taxon abundance. DAnIEL was run on 10 samples consisting simulated reads using denoising methods DADA2 and PIPITS and classification methods BLAST consensus (blast) and Naïve Bayes (nb). Difference of measured to true abundance was calculated for each sample and taxon. Furthermore, L2 norm of measured abundance profile to the true one was calculated for each sample. DADA2 in combination with BLAST yielded the most accurate results.

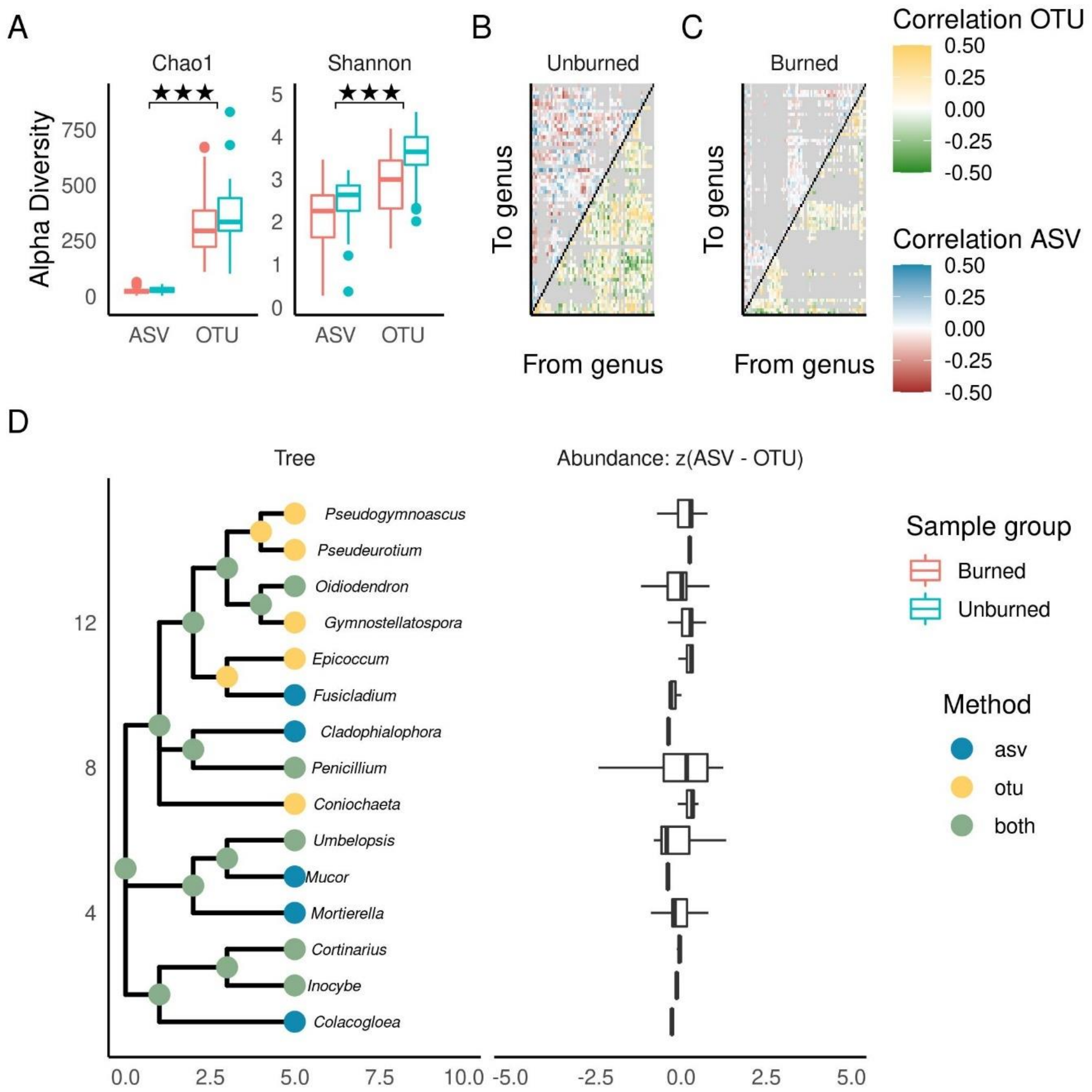

Fig S7: Comparison between ASV and OTU profiling in the soil case study. Overall, 70 out of the 73 significantly differentially abundant genera of the ASV method were also significant in the OTU method. **A**: Alpha diversity, **B/C**: SparCC correlation coefficients of co-abundant genera per sample group. Insignificant correlations are shown in grey. **D**: The phylogenetic tree shows, if a clade was present in any or both methods after keeping only the 10 taxa with the highest commutative abundance for each denoising method. Boxplots indicate the difference of total sum scaled abundances over the samples for each genus leaf in the tree. Values are z-scaled to variance of 1 and a mean of 0 for each taxon separately.

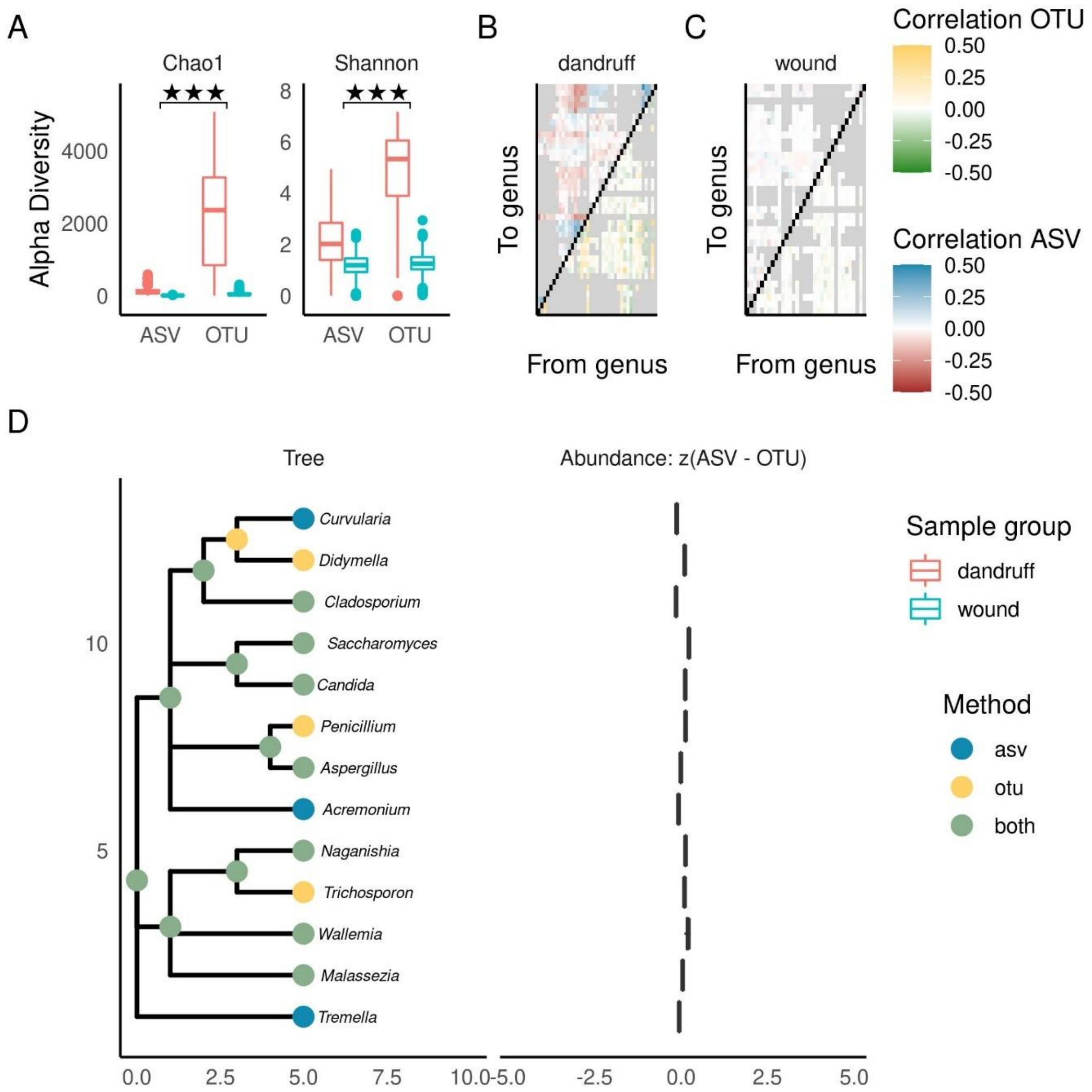

Fig S8: Comparison between ASV and OTU profiling in the human case study. Overall, 36 out of the 40 significantly differentially abundant genera of the ASV method were also significant in the OTU method. **A**: Alpha diversity, **B/C**: SparCC correlation coefficients of co-abundant genera per sample group. Insignificant correlations are shown in grey. **D**: The phylogenetic tree shows, if a clade was present in any or both methods after keeping only the 10 taxa with the highest commutative abundance for each denoising method. Boxplots indicate the difference of total sum scaled abundances over the samples for each genus leaf in the tree. Values are z-scaled to variance of 1 and a mean of 0 for each taxon separately.

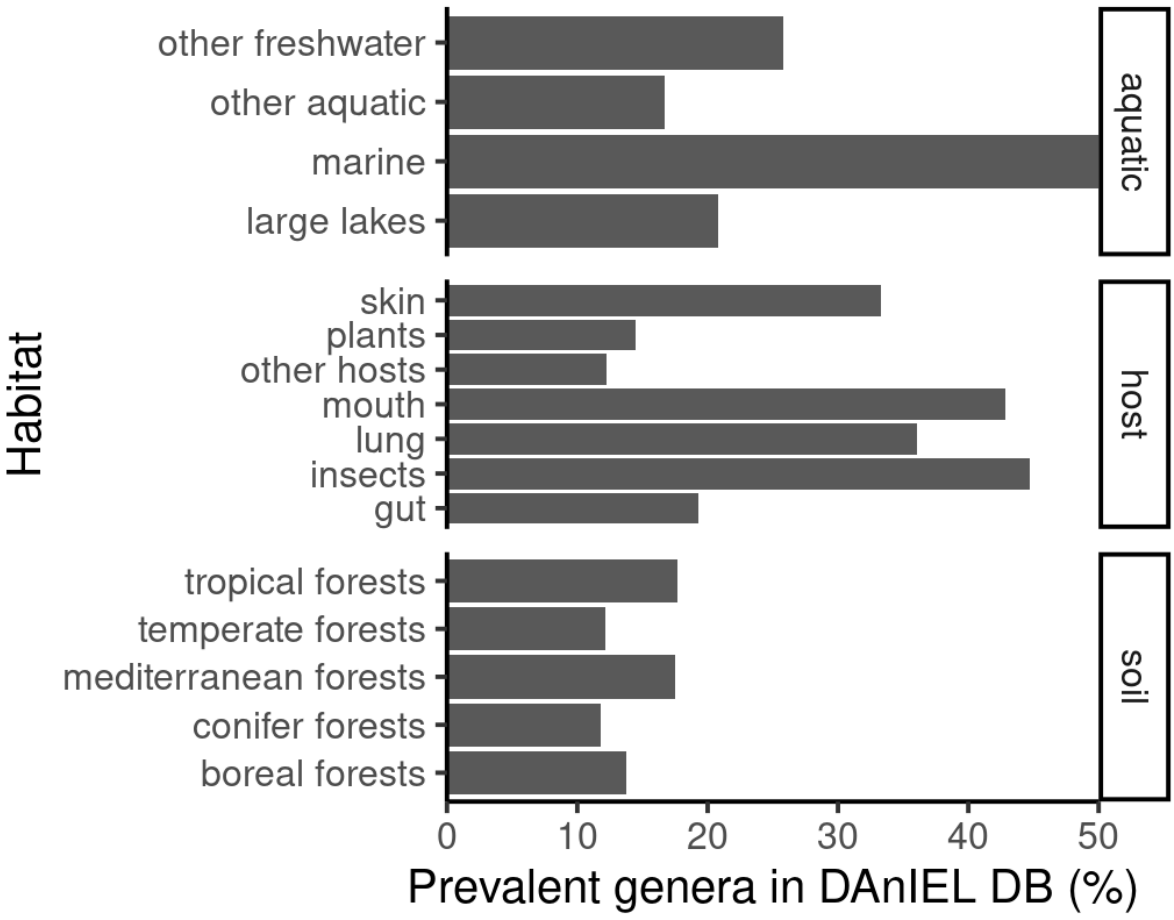

Fig S9: Generalisability of the DAnIEL database. The manual curated database is based on fungal species found in human samples. We analysed 30 projects from soil, aquatic and host related environments to see how many of the prevalent genera in these cohorts have already annotations in the database. NCBI bioprojects used:host: PRJEB11419, PRJEB23282, PRJEB32265, PRJNA241408, PRJNA271113, PRJNA295773, PRJNA359237, PRJNA418896, PRJNA473079, PRJNA478949, PRJNA496065, PRJNA525614, PRJNA550037
aquatic: PRJEB30970, PRJEB31590, PRJNA282687, PRJNA287840, PRJNA324410
soil: PRJNA263505, PRJNA378860, PRJNA406830, PRJNA415280, PRJNA432446, PRJNA450848, PRJNA492720, PRJNA517449, PRJNA526458, PRJNA528359, PRJNA561568, PRJNA606949
